# Valorization of Goat Blood: Hydrolysate Production, Identification, Stability, and Bioavailability upon Gastrointestinal Digestion of Peptides with Dual ACE and DPP-IV Inhibitory Properties

**DOI:** 10.3390/foods15101783

**Published:** 2026-05-18

**Authors:** Phanthipha Laosam, Yong Yue, Pichitpon Luasiri, Saranya Suwanangul, Nattapol Pongsamai, Daranee Chokchaichamnankit, Jisnuson Svasti, Chantragan Srisomsap, Mahmoud Rouabhia, Papungkorn Sangsawad

**Affiliations:** 1 School of Animal Technology and Innovation, Institute of Agricultural Technology, Suranaree University of Technology, Nakhon Ratchasima 30000, Thailand; laos.phanthipha@gmail.com (P.L.); yongyue1992@126.com (Y.Y.); pichitpon21831@gmail.com (P.L.); 2 Postharvest Technology and Innovation in Animal Unit, Institute of Agricultural Technology, Suranaree University of Technology, Nakhon Ratchasima 30000, Thailand; 3 Research and Development Institute, Suranaree University of Technology, Nakhon Ratchasima 30000, Thailand; 4 Program in Food Science and Technology, Faculty of Engineering and Agro-Industry, Maejo University, Chiang Mai 50290, Thailand; saranya_sw@mju.ac.th; 5 Suranaree University of Technology Hospital, Nakhon Ratchasima 30000, Thailand; nattienat.md@hotmail.com; 6 Laboratory of Biochemistry, Chulabhorn Research Institute, Bangkok 10210, Thailand; daranee@cri.or.th (D.C.); jisnuson@cri.or.th (J.S.); chantragan@cri.or.th (C.S.); 7 Applied Biological Sciences Program, Chulabhorn Graduate Institute, Bangkok 10210, Thailand; 8 Oral Ecology Research Group, Faculty of Dentistry, Laval University, Quebec City, QC G1V 0A6, Canada

**Keywords:** goat blood, bioactive peptides, angiotensin-converting enzyme (ACE) inhibition, dipeptidyl peptidase-IV (DPP-IV) inhibition, waste valorization, functional food

## Abstract

Goat blood, a major slaughterhouse by-product, was systematically valorized into dual-function bioactive peptides through an optimized four-step process. Four blood preparations—whole blood (HB), anticoagulant-treated blood (HBS), red blood corpuscles (BC), and plasma (PM)—were subjected to heat pretreatment (90 °C, 15 min) and enzymatic hydrolysis. Neutrase hydrolysis of heat-pretreated whole blood at 8% substrate concentration for 4 h (HBN-8) yielded optimal protein recovery (44.38%) with dual ACE (88.24%) and DPP-IV (81.13%) inhibition. Ultrafiltration enriched bioactive peptides in the ≤3 kDa fraction (DPP-IV: 87.8%; ACE: 65.5%). LC-MS/MS de novo sequencing identified 14 novel peptide sequences (4–9 amino acids), with the most potent SEC fraction showing IC_50_ values of 0.89 and 0.45 mg Leu eq./mL for DPP-IV and ACE inhibition, respectively. Critically, simulated gastrointestinal digestion enhanced rather than diminished bioactivity, with ACE inhibition increasing progressively to 60.91% at the intestinal phase, supported by predicted generation of bioactive fragments from parent sequences. Caco-2 assays confirmed peptide safety (100–1000 µg/mL) and demonstrated 10.47% transepithelial transport with retained dual inhibitory activities. This study establishes goat blood as a sustainable source of orally bioavailable, GI-stable peptides for the development of functional foods targeting hypertension and type 2 diabetes.

## 1. Introduction

In recent years, the global goat population has grown significantly, with Asia accounting for over 60% of global goat production [1]. This growth has led to increased goat meat processing and, consequently, larger volumes of processing by-products. Among these by-products, goat blood represents a particularly promising yet underutilized resource. Despite its high nutritional value, approximately 60% of goat blood from slaughterhouses is discarded as waste, creating both environmental concerns and missed economic opportunities [2].

Blood, a major by-product of slaughterhouses, is an excellent source of high-quality protein, containing approximately 17–20% protein, 80% water, various minerals, and other bioactive compounds [3,4]. Goat blood is distinguished by an amino acid profile particularly rich in histidine, branched-chain amino acids (BCAAs), and other essential amino acids that exceed WHO/FAO/UNU dietary reference values, properties that render it a particularly attractive substrate for dual-function peptide production [2]. In contrast, bovine blood-derived peptides have been characterized predominantly for antioxidant activity, with ACE and DPP-IV inhibition reported only as secondary outcomes [5]. Porcine blood hydrolysates, rich in leucine, lysine, and histidine, exhibit antioxidant, DPP-IV inhibitory, and anti-inflammatory activities [6], while duck blood peptides—abundant in hydrophobic and basic residues—have demonstrated antioxidant and ACE inhibitory activities without systematic evaluation of dual DPP-IV inhibition or oral bioavailability [7]. Likewise, chicken blood fractions enriched in aspartic acid and lysine have shown outstanding antioxidant performance [8]. Blood can be separated into two main fractions: blood corpuscles (20–40%) and plasma (60–80%) [9]. However, the specific fractionation strategy—including whether to process whole blood, anticoagulant-treated blood, red blood corpuscles, or plasma separately—substantially influences both hydrolysis efficiency and the bioactive profile of the resulting peptides, yet this has not been systematically compared for goat blood. Beyond bioactive peptide research, [10] animal blood and its fractions have established applications in food product development: plasma proteins are widely used as fat replacers, emulsifiers, and egg-white substitutes in meat and bakery products, whole blood and red cells are incorporated into traditional foods such as blood sausage (boudin noir, morcilla, black pudding) and blood-tofu, and blood-derived ingredients have been investigated for iron fortification in noodles, biscuits, and protein bars [4,9,11]. These existing applications confirm the technological feasibility of incorporating blood-derived ingredients into mainstream food matrices.

The development of functional foods with multiple bioactivities has gained significant attention in recent years, particularly those targeting interconnected metabolic disorders. Hypertension and diabetes frequently co-exist, affecting over 30% of adults globally [12]. This comorbidity imposes a compounding disease burden, as each condition accelerates the progression of the other through shared pathophysiological mechanisms including insulin resistance, renin–angiotensin system dysregulation, and endothelial dysfunction. Peptides exhibiting dual ACE and DPP-IV inhibitory activities are particularly valuable for developing novel functional foods, as they can simultaneously address both conditions through natural food-derived compounds [13,14]. ACE inhibition helps regulate blood pressure by preventing the formation of the vasoconstrictor angiotensin II, while DPP-IV inhibition enhances insulin secretion by protecting incretin hormones from degradation [15]. The discovery of natural peptides with these dual functions could provide safer alternatives to synthetic drugs, which often cause side effects such as dry cough and gastrointestinal discomfort [16]. The growing demand for clean-label, food-derived therapeutic ingredients positions animal blood hydrolysates—particularly from underutilized species such as goats—as strategically important yet largely untapped sources of dual-function bioactive peptides. Crucially, animal-source bioactive peptides have demonstrated direct in vivo and clinical relevance for these conditions: plasma- and blood-derived ACE-inhibitory peptides have produced significant antihypertensive effects in spontaneously hypertensive rat (SHR) models [17], while related food-derived peptides such as the lactotripeptides Val-Pro-Pro and Ile-Pro-Pro have lowered blood pressure in human clinical trials [10], underscoring the translational potential of blood-derived peptides for managing hypertension and type 2 diabetes.

Enzymatic hydrolysis represents a particularly attractive technique for converting underutilized animal blood into high-value products. The process’s efficiency is measured by the degree of hydrolysis (DH), which significantly influences the nutritional and functional properties of the resulting protein hydrolysate [18,19]. The optimization of hydrolysis parameters—including substrate concentration, enzyme type, temperature, time, and pH—is crucial for developing economically viable and biologically active peptides. Beyond hydrolysis optimization, translating in vitro bioactivity into physiological relevance requires demonstrating peptide stability during gastrointestinal (GI) digestion and successful transepithelial absorption, two critical bottlenecks that many food-derived peptide studies fail to address [5,7]. The size and sequence of peptides significantly influence their bioavailability and biological activities, particularly their ability to inhibit DPP-IV and ACEs [16,20]. Critically, GI digestion does not always attenuate peptide bioactivity; in some systems, digestive proteolysis generates smaller, more potent bioactive fragments from larger precursor sequences, a phenomenon of considerable functional significance that merits systematic investigation for goat blood hydrolysates [5,7].

Building upon our research group’s successful development of bioactive peptides from duck blood [7] and bovine blood [5], this study extends the technology to goat blood processing by-products. While previous work established the feasibility of blood-derived bioactive peptides, goat blood remains unexplored in terms of (i) systematic comparison of blood fractions as hydrolysis substrates, (ii) identification of novel peptide sequences through LC-MS/MS de novo sequencing, (iii) evaluation of bioactivity enhancement or attenuation during standardized INFOGEST simulated GI digestion, and (iv) quantification of transepithelial transport and retained dual inhibitory activity using Caco-2 cell monolayers. The established methodology for heat treatment and enzymatic hydrolysis from our previous work provided a foundation for optimizing peptide production from goat blood, while targeting additional bioactivity through DPP-IV inhibition. Addressing these gaps is essential to establish whether goat blood hydrolysates can bridge the critical divide between in vitro bioactivity and in vivo therapeutic relevance—a prerequisite for their credible application as functional food ingredients.

Although bioactive peptides from bovine, porcine, and duck blood have been previously reported, those studies were largely limited to single-target activities—predominantly antioxidant—and rarely evaluated peptide gastrointestinal stability or transepithelial bioavailability. The present work is therefore the first to combine fraction-level substrate optimization, LC-MS/MS de novo peptide identification, and coupled GI-stability and Caco-2 transport assessment specifically for dual ACE/DPP-IV inhibitory peptides from goat blood—a histidine- and BCAA-rich slaughterhouse by-product that has remained largely untapped.

Therefore, this study aimed to develop and characterize bioactive peptides from goat blood with dual ACE and DPP-IV inhibitory properties through a systematic four-step approach: (i) comparative characterization of four blood fractions (whole blood, anticoagulant-treated blood, red blood corpuscles, and plasma) as hydrolysis substrates; (ii) stepwise optimization of enzyme type, substrate concentration, and hydrolysis time; (iii) purification and LC-MS/MS-based identification of novel dual-function peptide sequences; and (iv) evaluation of peptide stability and bioactivity during simulated GI digestion and transepithelial transport across Caco-2 cell monolayers. The findings of this study are expected to provide a scientifically grounded, practically applicable framework for valorizing goat slaughterhouse blood waste into high-value functional ingredients targeting the globally prevalent comorbidity of hypertension and type 2 diabetes.

## 2. Materials and Methods

### 2.1. Chemicals and Reagents

Two food-grade proteases, Papain (6000 USP U/mg) and Neutrase (0.8 AU-N/g), were sourced from Brenntag Ingredients (Thailand) Company Limited, Bangkok, Thailand. The following reagents were obtained from Sigma-Aldrich (St. Louis, MO, USA): angiotensin I-converting enzyme (ACE) isolated from rabbit lungs, N-[3-(2-Furyl) acryloyl]-Phe-Gly-Gly (FAPGG), dipeptidyl peptidase IV (DPP-IV), Gly-Pro p-nitroanilide hydrochloride, 2,4,6-trinitrobenzene sulphonic acid (TNBS), 6-hydroxy-2,5,7,8-tetramethyl chroman-2-carboxylic acid (Trolox), 2,4,6-tris(2-pyridyl)-s-triazine (TPTZ), ferric(III) chloride, ferrozine, acetonitrile (ACN), trifluoroacetic acid (TFA), pepsin (800 units/mg solid, derived from porcine gastric mucosa; P7000), and pancreatin (5.99 U TAME/mg solid, derived from porcine pancreas, 8 × USP). All remaining chemicals and reagents used throughout the study were of analytical grade.

### 2.2. Chemical Composition Analysis of Goat Blood

#### 2.2.1. Preparation of Blood Samples

Fresh goat blood samples were obtained from a commercial slaughterhouse (Goat-Sheep Slaughterhouse, Thailand). Blood was collected from healthy goats (breed: mixed Boer and Anglo-Nubian; age: 6–8 months; both sexes) at the time of slaughter, with a total volume of approximately 15 L pooled from ~10–15 animals per batch. Samples were transported to the laboratory on ice within 2 h and processed immediately. Two types of blood samples were prepared: whole blood without anticoagulant (HB) and whole blood with anticoagulant (sodium citrate, HBS). For HBS preparation, fresh blood was immediately mixed with 1% sodium citrate (*w*/*v*) at a 1:100 (g/100 mL) ratio to prevent coagulation. The HBS samples were then centrifuged (5000× *g*, 4 °C, 10 min) to separate blood corpuscles (BC) from plasma (PM). All samples (HB, HBS, BC, and PM) were stored at 4 °C until further analysis.

#### 2.2.2. Proximate and Mineral Composition Analysis

Proximate composition, including moisture, crude protein, and ash content, was determined according to the official methods of AOAC [21], while crude fat content was measured following the procedure described by Folch et al. [22]. For mineral analysis, each goat blood sample (500 mg) was digested by mixing with 8 mL of an acid mixture consisting of 65% nitric acid and 30% hydrogen peroxide at a 3:1 (*v*/*v*) ratio. The mixture was subjected to wet digestion at 110–130 °C for 4 h until complete solubilization was achieved. The resulting digest was filtered through Whatman No. 1 filter paper, quantitatively transferred into a volumetric flask, and diluted to a final volume of either 10 or 25 mL with deionized water. Mineral concentrations were subsequently quantified by inductively coupled plasma optical emission spectrometry (ICP-OES; PerkinElmer PinAAcle 900F, Atomic Absorption Spectrophotometer, PerkinElmer, Inc., Waltham, MA, USA) following the approach of Paranthaman et al. [23] with minor modifications, using detection wavelengths of 248.20, 213.86, and 589.59 nm for Fe, Zn, and Na, respectively. All mineral concentrations were expressed as mg per 100 mL of sample.

#### 2.2.3. Amino Acid Composition Analysis

Amino acid profiles of goat blood samples were analyzed using the protocol by Sangsawad et al. [14]. Freeze-dried blood samples (0.05 g) were hydrolyzed with 6 M Hydrochloric acid (HCl) (10 mL), mixed with a vortex Mixer (Vortex genie 2, Scientific Industries, Bohemia, NY, USA), and placed in a heating bath at 110 °C for 24 h. The hydrolyzed samples were adjusted to 10 mL with DI type I. The diluted samples were treated with 0.1 M ammonium formate and filtered through a 0.2 μm syringe filter into a vial for amino acid analysis using LC-MS/MS (LCMS-8060, Shimadzu, Kyoto, Japan). For tryptophan analysis, the freeze-dried blood samples (0.05 g) were hydrolyzed with 4.2 M sodium hydroxide (NaOH) (10 mL). Their amino acid composition was determined by LC-MS/MS (LCMS-8060, Shimadzu, Japan) using Intrada Amino Acid (Pure Spherical Silica) (50 mm × 3 mm, 3 µm) column. Samples (1 µL) were injected into an LC-MS/MS using a mobile phase of solvent A (acetonitrile with 0.1% formic acid) and solvent B (0.1 M ammonium formate). The separation gradient was performed as follows: 0–3 min (14% B), 3–10 min (100% B), 10–15 min (14% B) at a flow rate of 0.6 mL/min. The amino acids and dipeptides composition and content were calculated from the standard curve of amino acids.

### 2.3. Optimization of Enzymatic Hydrolysis Conditions

#### 2.3.1. Effects of Enzyme Type and Thermal Pretreatment

Raw and heat-treated (90 °C for 15 min before hydrolysis) goat blood samples (HB, HBS, BC, and PM) were hydrolyzed using two commercial proteases: Neutrase and Papain. Each enzyme was added at 5% of the protein content (within the product recommendation). This 5% (*w*/*w* of substrate protein) ratio was selected to remain within the manufacturer-recommended operating range for both food-grade enzymes and to align with established protocols for animal blood protein hydrolysis at laboratory scale, including our previous studies on duck blood [7] and bovine blood [5]. The hydrolysis conditions were: Neutrase (pH 7.0, 50 °C) and Papain (pH 7.0, 65 °C). The reaction was carried out for 4 h with continuous shaking. The most effective enzyme was selected based on the degree of hydrolysis, total α-amino content, protein recovery, and enzyme inhibitory activities.

#### 2.3.2. Effect of Substrate Protein Concentration

The optimal substrate form (raw or heated) and the enzyme determined in 2.3.1 were used to study the effect of protein concentration. Three concentrations (4%, 8%, and 12% *w*/*v*) were prepared by dissolving the substrate in deionized water. This range was chosen to bracket the typical operating window for enzymatic hydrolysis of animal blood proteins, where concentrations below 4% are economically inefficient and those above 12% commonly experience reduced enzyme accessibility due to increased substrate viscosity and aggregation. Hydrolysis was performed using the selected enzyme under previously optimized conditions. The optimal concentration was determined based on the degree of hydrolysis and bioactive peptide yield.

#### 2.3.3. Effect of Hydrolysis Time

Using the optimal conditions from previous steps (substrate form, enzyme type, and concentration), the effect of hydrolysis time was studied. The reaction was monitored at 0, 2, 4, 6, 8, and 10 h. During hydrolysis, pH was adjusted every 2 h to maintain optimal conditions. Samples were taken at each point for analysis of α-amino content, bioactive properties, and molecular weight distribution.

### 2.4. Hydrolysate Characterization

#### 2.4.1. Degree of Hydrolysis Determination

The degree of hydrolysis was assessed following the trinitrobenzenesulfonic acid (TNBS) colorimetric procedure described by Adler-Nissen [24], with minor modifications. Briefly, protein hydrolysate samples (10 µL) were combined with 0.2125 M sodium phosphate buffer, pH 8.2 (100 µL), and 0.05% TNBS reagent (50 µL) in a 96-well microplate. L-leucine was used as the standard for expressing free alpha-amino group content. The reaction mixture was incubated at 45 °C, and absorbance was recorded at 420 nm over 30 min using a microplate reader (Varioskan LUX, Thermo Scientific, Vantaa, Finland). The percentage degree of hydrolysis (DH, %) was calculated according to the following equation:

Degree of hydrolysis (DH, %) = [(α_A1_-α_A0_)/α_At_] × 100 (1)

where α_A0_ and α_A1_ denote the free alpha-amino content at the initial timepoint (0 h) and at each subsequent measurement interval, respectively, and α_At_ represents the total alpha-amino content obtained from complete hydrolysis of the sample (120 °C, 6 N HCl, 24 h). Throughout this study, peptide concentration is expressed as mg L-leucine equivalents per mL (mg Leu eq./mL), calculated from the TNBS-derived α-amino group content using L-leucine as the calibration standard. This unit is the conventional metric for hydrolysate-level peptide quantification and is consistently applied to all subsequent measurements, including total α-amino content, IC_50_ values, and assay concentrations.

#### 2.4.2. DPP-IV and ACE Inhibitory Activities Analysis

The DPP-IV inhibition of the peptide was measured according to Lacroix and Li-Chan [25] technique. The reaction mixture was performed in a 96-well microplate. The sample (20 µL) was mixed with 10 µL of DPP-IV (0.01 U/mL) and preincubated for 5 min at 37 °C. Afterward, 50 µL of the substrate Gly-Pro-p-nitroanilide (30 mM) was added, and the mixture was incubated for 30 min at 405 nm using a microplate reader (Varioskan LUX, Thermo Scientific, Vantaa, Finland). Deionized (DI) water was used as the blank for the sample solutions. DPP-IV activity was defined as the following equation:

DPP-IV inhibition (%) = [(Slope _(positive control)_-Slope _(test sample)_)/(Slope _(positive control)_)] × 100 (2)

The ACE inhibition of the peptide was measured according to the method of Luasiri et al. [26]. The reaction mixture was performed in a 96-well microplate. The sample (20 µL) was mixed with 10 µL of ACE (1 mU/mL) and preincubated for 5 min at 37 °C. Afterward, 80 µL of substrate FAPGG (0.5 mM) was added, and the mixture was incubated for 30 min at 340 nm using a microplate reader (Varioskan LUX, Thermo Scientific, Vantaa, Finland). Deionized (DI) water was used as the blank for the sample solutions. The ACE activity was represented as the reaction rate (ΔA/min), and the inhibitory activity was estimated using the following formula:

ACE inhibition (%) = (Slope _(positive control)_-Slope _(test sample)_)/(Slope _(positive control)_) × 100 (3)

#### 2.4.3. Peptide Molecular Weight (MW) Distribution Analysis

The molecular weight distribution of the peptides was measured using size-exclusion chromatography (SEC), with a slight modification by Laosam et al. [7]. The sample, 100 µL of 1.0 mg Leu/mL, was injected into a Fast Protein Liquid Chromatography (FPLC, AKTA PURE, GE Healthcare, Uppsala, Sweden) with a Superdex Peptide 10/300 GL column (10 mm × 300 mm, GE Healthcare, Piscataway, NJ, USA). UV 215 nm was used to monitor the elution of 0.1% trifluoroacetic acid (TFA) in 30% acetonitrile (ACN) at a flow rate of 0.5 mL/min. The molecular weight was calculated using a calibration curve generated from the following reference standards: Cytochrome C (12,000 Da), Aprotinin (6512 Da), AGNQVLNLQADLPK (1480 Da), NTFLFFK (916 Da), DLE (375 Da), and Tryptophan (204 Da).

### 2.5. Fractionation, Purification, and Identification of Bioactive Peptides

#### 2.5.1. Ultrafiltration-Based Separation

The goat blood hydrolysate peptides obtained from Neutrase hydrolysis of 8% HB protein substrate (HBN-8) at 4 h were fractionated by ultrafiltration using UF membranes Vivaspin^®^ 20 (Sartorius, Göttingen, Germany) with molecular weight cut-offs (MWCOs) of 10 and 3 kDa, respectively. The obtained fractions were designated as follows: ≥10, 10–3, and ≤3 kDa. After that, the peptide fractions were collected before analyzing the α-amino group and biological activity. We calculated the yield of peptides (%) using the equation

Peptide recovery (%) = (α-amino in fractions)/(α-amino of initial sample (HBN-8)) × 100(4)

#### 2.5.2. Size Exclusion Chromatography (SEC) Purification

The peptides with size ≤ 3 kDa (UF3) exhibiting the highest bioactivity were purified by size-exclusion chromatography (SEC) with a slight modification of the method described by Sangsawad et al. [14], using Fast Protein Liquid Chromatography (FPLC; AKTA explorer, GE Healthcare, Uppsala, Sweden). A sample of 100 µL of peptides was applied to a Superdex Peptide 10/300 GL column (10 mm × 300 mm, GE Healthcare, Piscataway, NJ, USA). The elution was performed using DI water (A) and 30% acetonitrile (ACN) + 0.1% trifluoroacetic acid (TFA) (B) at a flow rate of 0.8 mL/min. The elution stepwise was started with 100% of mobile phase B for 3–3.75 mL, 2.5% for 3.75–6 mL, 100% B for 6–6.75 mL, 2.5% B for 6.75–8 mL, and 100% B for 8–14 mL. The fractions were collected and lyophilized (Freeze Dryers, CHRIST/Gamma 2–16 LSC, Martin Christ Gefriertrocknungsanlagen GmbH, Osterode am Harz, Germany) to remove the mobile phase before analysis of the α-amino group and biological activity. SEC calibration standards: Cytochrome C (12,000 Da), Aprotinin (6512 Da), AGNQVLNLQADLPK (1480 Da), NTFLFFK (916 Da), DLE (375Da) and Tryptophan (204 Da). We calculated the yield of peptides (%) using the equation

Peptide recovery (%) = [(α-amino in fractions)/(α-amino of initial sample (≤3 kDa))] × 100(5)

#### 2.5.3. Peptide Sequencing by LC-MS/MS and in Silico Analysis

Peptides from the F3 fraction were identified by ESI-ion trap mass spectrometry (AmaZon SL, Bruker Daltonics, Bremen, Germany) following Laosam et al. [7]. Separation was performed on an AdvanceBio Peptide Plus column using a water–acetonitrile gradient with 0.1% formic acid, and mass spectra were acquired at m/z 50–1500 in positive-ion mode at 1.6 kV ESI. Sequences were determined by de novo sequencing using PEAKS Studio 10.0 (Bioinformatics Solutions Inc., Waterloo, ON, Canada) (score > 90%). Identified peptides were subjected to in silico gastrointestinal digestion (pepsin, trypsin, and chymotrypsin) via the DFBP database (http://www.cqudfbp.net/, accessed on 1 March 2026), and their ACE and DPP-IV inhibitory activities were evaluated using the BIOPEP database https://biochemia.uwm.edu.pl/biopep/start_biopep.php, accessed on 1 March 2026). Physicochemical properties, including molecular weight and isoelectric point, were calculated using PepDraw (http://pepdraw.com/, accessed on 1 March 2026). Toxicity was predicted by ToxinPred (https://webs.iiitd.edu.in/raghava/toxinpred/, accessed on 1 March 2026), bioactivity probability by PeptideRanker (http://bioware.ucd.ie/~compass/biowareweb, accessed on 1 March 2026), and cell-penetrating potential by CPPpred (https://peptide.ucd.ie/cpppred/, accessed on 1 March 2026).

### 2.6. Gastrointestinal Stability Assessment

The gastrointestinal stability of the 4 h HBN-8 hydrolysate was investigated through an in vitro digestion model adapted from the protocol of Minekus et al. [27], with minor procedural modifications. In the gastric phase, the peptide solution (40 mg/mL) was combined with Simulated Gastric Fluid (SGF) in a 1:1 (*v*/*v*) proportion, and the pH of the resulting mixture was adjusted to 3.0. Pepsin (800 U/mg solid; 0.5 mL) was subsequently introduced together with CaCl_2_ (75 µM) to yield a final enzymatic activity of 2000 U/mL. The system was incubated for 2 h in a shaking water bath (37 °C, 150 rpm), after which gastric digestion was terminated by neutralizing the pH to 7.0.

For the intestinal phase, the neutralized gastric digest was mixed with Simulated Intestinal Fluid (SIF) at a 1:1 (*v*/*v*) ratio, followed by the addition of pancreatin (0.5 mL) and CaCl_2_ (0.3 mM) to achieve a final activity of 100 U/mL. Incubation was carried out under the same shaking conditions (37 °C, 150 rpm) for an additional 2 h. Enzymatic reactions were inactivated by heating at 95 °C for 15 min, followed by immediate cooling on ice for 5 min. The mixture was then centrifuged at 10,000× *g* for 10 min at 4 °C, and the resulting supernatants were collected for further analysis. Free α-amino groups in the digesta were quantified by the TNBS assay following the procedure of Adler-Nissen [24]. To evaluate the impact of simulated digestion on bioactivity, both ACE and DPP-IV inhibitory activities were determined in the pre- and post-digestion samples.

### 2.7. In Vitro Bioavailability Assessment of the HBN-8 Peptide Derived After GI Digestion Using Caco-2 Cell Monolayers

The experiment followed the procedure of Luasiri et al. [26]. Caco-2 cells (ATCC No. HTB-37), obtained from the American Type Culture Collection (Rockville, MD, USA), were used as an in vitro model to evaluate peptide bioavailability. Cells at passages 20–30 were cultured in EMEM supplemented with fetal bovine serum (FBS) and antibiotics at 37 °C in a humidified atmosphere containing 5% CO_2_ for 21 days. The study first evaluated the cytotoxicity of GI peptides derived from HBN-8 at 4 h, at concentrations ranging from 0 to 1000 µg/mL, using an MTT assay after 24 h incubation, in which cell viability was measured as formazan production at 570 nm. For the transport study, Caco-2 cells were grown on culture inserts for 21 days, and only monolayers showing TEER values above 250 Ω U/cm^2^ were selected. The experiment involved adding peptide samples (500 µg/mL) to the apical side, monitoring peptide content at 0 and 2 h in the apical compartment, and then at 2 h in the basal compartment. Bioavailability was calculated from protein content using a UV assay at 215 nm in both compartments, and the transported peptides were further analyzed for their ACE and DPP-IV inhibitory activities, expressed as IC_50_ values.

### 2.8. Statistical Analysis

All experiments were performed as three independent biological replicates (*n* = 3), with each measurement carried out in technical triplicate. Quantitative results are expressed as mean ± standard deviation (SD). Statistical analyses were conducted using SPSS Statistics 16.0 (SPSS Inc., Chicago, IL, USA). Differences among treatment groups were evaluated by one-way analysis of variance (ANOVA), followed by Duncan’s multiple range test for post hoc pairwise comparisons. Statistical significance was set at *p* < 0.05. Different lowercase letters (a, b, c, …) above bars in figures or values in tables indicate statistically significant differences within the same column or treatment group. Error bars in all figures represent ± SD of three independent replicates, and ±SD values are reported alongside means in all tables.

## 3. Results and Discussion

### 3.1. Chemical Composition of Goat Blood

#### 3.1.1. Proximate and Mineral Composition

The analysis of different goat blood fractions revealed distinct compositional profiles that significantly influence their potential as substrates for enzymatic hydrolysis (Table 1). Blood corpuscles (BC) demonstrated the highest protein content (35.7%), nearly twice that of whole blood (HB; 20.5%) and approximately 5-fold higher than plasma (PM; 6.71%). This protein concentration gradient suggests BC as a particularly rich source for bioactive peptide production [4]. Their mineral distributions further evidenced the compositional uniqueness of each fraction. HB exhibited the highest sulfur content (418 ± 10 mg/100 mL), followed by BC (400 ± 34 mg/100 mL) and HBS (310 ± 10 mg/100 mL), while BC exhibited elevated phosphorus levels (480 ± 6 mg/100 mL). PM displayed notably high sodium concentrations (655 ± 23 mg/100 mL), which can be attributed to the sodium citrate anticoagulant being carried into the plasma fraction following centrifugation [9]. The elevated ash content in PM (1.91%) compared to other fractions (1.05–1.61%) is consistent with the higher mineral accumulation observed in PM. The fat content remained consistently low across all fractions (<0.3%), suggesting minimal interference with protein extraction and subsequent enzymatic hydrolysis processes. This compositional variation between fractions—particularly in protein content and mineral distribution—indicates their potential to yield distinct peptide profiles upon enzymatic hydrolysis. The higher protein content in BC suggests it may generate a more diverse range of bioactive peptides, while the distinct mineral compositions could influence enzyme activity and peptide functionality.

#### 3.1.2. Amino Acid Composition

Comparative analysis of amino acid profiles across four goat blood fractions (HB, HBS, BC, and PM) revealed distinct compositional patterns (Table 2). Essential amino acids (EAAs) are indispensable for human health as they cannot be synthesized endogenously and must be obtained through diet, playing critical roles in protein synthesis, tissue repair, and metabolic regulation [28,29]. The total EAA content varied among fractions, with PM showing the highest concentration (56.31%), followed by HBS (49.92%), HB (49.71%), and BC (47.27%), all substantially exceeding the WHO/FAO/UNU reference requirements, suggesting that all fractions represent high-quality protein sources.

Among individual EAAs, L-Histidine was remarkably elevated across all fractions, with PM containing the highest level (12.12 g/100 g protein), far exceeding the WHO/FAO/UNU reference value (1.50 g/100 g protein). Histidine is particularly important as a precursor for the synthesis of carnosine and anserine and plays a central role in growth, tissue repair, and immune function [30]. L-Isoleucine was consistently high across all fractions (7.13–7.96 g/100 g protein), substantially surpassing the recommended reference (3.00 g/100 g protein), which is nutritionally significant given its role in energy metabolism and immune regulation. L-Lysine, an EAA critical for collagen synthesis and calcium absorption [31], was highest in PM (5.38 g/100 g protein) compared to BC (2.93 g/100 g protein), HBS (3.95 g/100 g protein), and HB (3.75 g/100 g protein), indicating superior protein quality of the plasma fraction in this regard. Regarding non-essential amino acids (non-EAAs), BC exhibited the highest total non-EAA content (52.73%), followed by HB (50.29%), HBS (49.67%), and PM (43.69%), suggesting that BC contains a particularly rich and diverse amino acid matrix as a substrate for hydrolysis [32]. HB and BC showed notably high levels of L-Alanine (HB: 10.83, BC: 9.63 g/100 g protein) and L-Serine (BC: 10.15, HB: 9.59 g/100 g protein). L-Glutamic acid was highest in HBS (9.70 g/100 g protein), slightly exceeding the WHO/FAO/UNU reference value (9.34 g/100 g protein), while HB (6.44 g/100 g protein) also approached this reference level. These amino acids are structurally relevant as precursors for numerous bioactive sequences and contribute to the overall nitrogen profile available for peptide bond cleavage during enzymatic hydrolysis.

The branched-chain amino acids (BCAAs—leucine, isoleucine, and valine) are of particular nutritional importance due to their roles in muscle protein synthesis and glucose homeostasis [33]. HBS exhibited the highest total BCAA content (20.58%), followed by BC (18.63%), HB (18.15%), and PM (17.40%). The high BCAA content across all fractions supports the nutritional value of goat blood proteins and their potential for applications in sports nutrition and metabolic health [34]. Overall, these compositional differences confirm that each goat blood fraction possesses a distinct amino acid matrix that would influence the nature and diversity of peptides released upon enzymatic hydrolysis [35]. The rich and varied amino acid profiles—particularly the abundance of EAAs, BCAAs, and structurally relevant non-EAAs—establish these fractions as promising substrates for producing peptides with targeted nutritional and functional properties.

### 3.2. Optimization of Enzymatic Hydrolysis

#### 3.2.1. Effect of Thermal Pretreatment and Enzyme Type on Hydrolysis Efficiency

The impact of thermal pretreatment (90 °C, 15 min) on enzymatic hydrolysis efficiency was evaluated across four goat blood fractions (HB, HBS, BC, and PM) using Neutrase and Papain, with results assessed through degree of hydrolysis (DH), protein recovery, and dual enzyme inhibitory activities (Table 3). In the absence of heat pretreatment, both enzymes showed modest hydrolysis efficiency across all fractions. DH values for raw samples ranged from 5.29 to 15.60% (Neutrase) and 4.57–15.27% (Papain), with no consistent superiority of either enzyme across all fractions. Notably, Neutrase achieved higher DH for RHB (15.60% vs. 14.92%) and RPM (5.29% vs. 4.57%), while Papain performed better for RHBS (15.27% vs. 14.52%) and RBC (12.12% vs. 10.88%). Protein recovery in raw samples was correspondingly limited (9.21–32.04%), and bioactive inhibitory activities remained relatively low, particularly for ACE inhibition by Neutrase (18.13–41.75%).

Heat pretreatment markedly enhanced hydrolysis efficiency across all fractions and both enzymes, consistent with the well-established role of thermal denaturation in unfolding protein structures and exposing peptide bonds to enzymatic attack [36]. DH values increased substantially after heating, with HHB-Neutrase rising from 15.60% to 18.53% and HBC-Papain from 12.12% to 15.61%. Protein recovery similarly improved, with HHB-Neutrase achieving 40.28% versus 30.60% for the raw sample. Most strikingly, heat pretreatment produced dramatic enhancements in dual bioactive properties. ACE inhibitory activity of HHB-Neutrase increased from 24.9% (raw) to 88.97% (heated), and DPP-IV inhibition rose from 27.09% to 83.77%. Similarly, HBC-Papain showed the highest overall DPP-IV and ACE inhibition among all heated fractions (89.04% and 94.30%, respectively). These improvements are consistent with findings by Nongonierma and FitzGerald [37] regarding the optimization of bioactive peptide production through thermal pretreatment, and by Arrutia et al. [36], who demonstrated that heat-induced protein unfolding maximizes enzyme accessibility to cleavage sites.

Although HPM fractions showed high ACE inhibition (87.59% for Neutrase), their markedly low DH (7.27%) and protein recovery (13.53%) render them less suitable for scalable peptide production. Based on the combined criteria of DH, protein recovery, and dual bioactive properties, three substrate–enzyme combinations were selected for further optimization: heat-treated whole blood with Neutrase (HBN), heat-treated whole blood with Papain (HBP), and heat-treated blood corpuscles with Papain (BCP).

#### 3.2.2. Optimization of Substrate Concentration on Hydrolysis Efficiency

The effect of substrate protein concentration (4%, 8%, and 12% *w*/*v*) on hydrolysis efficiency and bioactive properties was systematically investigated for the three selected combinations (HBN, HBP, and BCP) identified in the previous section (Table 4). Contrary to the common assumption that lower substrate concentration always yields higher DH due to greater enzyme–substrate accessibility, the 8% concentration consistently produced the highest DH across all three combinations (HBN: 26.47%, HBP: 21.08%, BCP: 18.57%), outperforming both 4% and 12% conditions. This suggests that at 4%, although accessibility is high, the absolute amount of releasable peptides per reaction volume is limiting. In contrast, the 8% concentration provides an optimal balance between substrate availability and enzyme efficiency.

Protein recovery followed the same trend, with the 8% condition yielding the highest values for all combinations. HBN-8 demonstrated the superior protein recovery (45.91%) compared to HBP-8 (41.67%) and BCP-8 (35.59%), further supporting HBN as the most productive substrate–enzyme pairing identified in Section 3.2.1. For dual bioactive properties, HBN-8 exhibited strong DPP-IV inhibition (83.86%) and the highest ACE inhibition among all HBN concentrations (86.79%). Notably, BCP at 4% showed the highest DPP-IV inhibition overall (88.84%), and BCP-8 also maintained high ACE inhibition (87.09%). However, given that HBN-8 delivered superior protein recovery alongside consistently high dual inhibitory activities, it was considered the most balanced and scalable candidate for further optimization.

The decline in both DH and bioactive properties at 12% protein concentration across all combinations can be attributed to increased substrate viscosity and protein aggregation, which restrict enzyme mobility and reduce effective enzyme–substrate contact [38]. Furthermore, the accumulation of peptide products at higher substrate loads may induce product inhibition, competitively interfering with enzyme–substrate binding and reducing overall hydrolysis efficiency [38]. This concentration-dependent reduction in performance is consistent with our previous findings on duck blood hydrolysates [7] and bovine blood hydrolysates [5], where excessive substrate concentration similarly compromised enzymatic efficiency.

Based on the combined evaluation of DH, protein recovery, and dual ACE/DPP-IV inhibitory activities, HBN-8 was selected as the optimal condition for subsequent hydrolysis time optimization, offering the most favorable balance of production efficiency and bioactive functionality.

#### 3.2.3. Effect of Hydrolysis Time on Peptide Yield and Bioactivity

**Figure 1:**
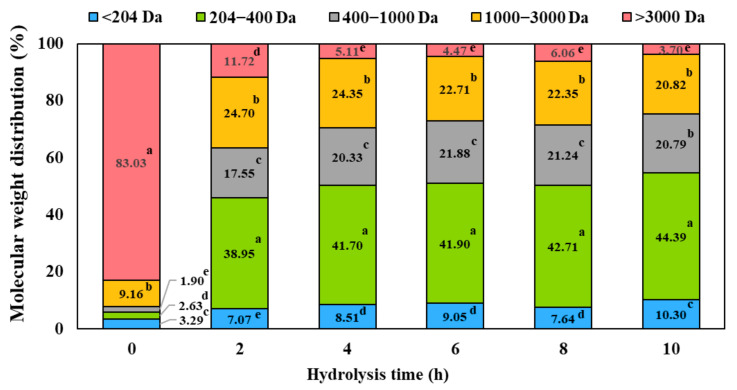
Molecular weight distribution (%) of peptides in goat blood hydrolysate (HBN-8) at hydrolysis times of 0–10 h. Samples were analyzed at 10 mg Leu eq./mL. The MW ranges (>3000, 3000–1000, 1000–400, 400–204, and <204 Da) were defined by SEC calibration with Cytochrome C (12,000 Da), Aprotinin (6512 Da), AGNQVLNLQADLPK (1480 Da), NTFLFFK (916 Da), DLE (375 Da), and Tryptophan (204 Da). Different letters above the bars indicate statistically significant differences within each molecular weight fraction (*p* < 0.05).

Following the selection of HBN-8 as the optimal substrate–enzyme combination, hydrolysis time was systematically optimized by evaluating HBN-8 at 0, 2, 4, 6, 8, and 10 h. Results for total α-amino acid content, DH, protein recovery, and dual bioactive activities are presented in Table 5, and the MW distribution profiles are shown in Figure 1. Total α-amino acid content, DH, and protein recovery all increased rapidly during the first 4 h of hydrolysis, reaching 298.80 mg Leu eq./mL, 28.13%, and 44.38 g/g protein, respectively. Beyond 4 h, further increases in these parameters were numerically marginal and statistically non-significant (*p* > 0.05), with values at 6, 8, and 10 h ranging from 301.12 to 314.10 mg Leu eq./mL, 29.88–32.34%, and 46.53–48.56 g/g protein, indicating that the hydrolysis reaction had effectively plateaued. Dual bioactive properties followed the same pattern, with DPP-IV and ACE inhibitory activities peaking at 4 h (81.13% and 88.24%, respectively) and showing no statistically significant improvement at extended times (*p* > 0.05), confirming 4 h as the inflection point for both productivity and bioactivity.

The MW distribution profiles (Figure 1) provided complementary mechanistic insights into the hydrolysis progression. At 0 h, the heat-pretreated sample was dominated by large proteins and peptides (>3000 Da; 83.03%), with minor proportions of 204–400 Da (9.16%), <204 Da (3.29%), 1000–3000 Da (1.90%), and 400–1000 Da (2.63%) fractions, reflecting the largely intact protein structure following thermal denaturation before enzymatic attack. After 2 h of Neutrase hydrolysis, the >3000 Da fraction decreased dramatically to 11.72%. In contrast, the 1000–3000 Da (24.70%), 400–1000 Da (17.55%), and 204–400 Da (38.95%) fractions increased markedly, indicating rapid and extensive enzymatic cleavage of large proteins into medium- and small-sized peptides.

The distribution stabilized substantially after 4 h, with the 204–400 Da fraction remaining dominant (41.70–44.39% from 4 to 10 h), followed by the 400–1000 Da fraction (20.33–20.79%) and the 1000–3000 Da fraction (20.82–24.35%). The >3000 Da fraction continued to decline gradually (5.11% at 4 h to 3.70% at 10 h), while the <204 Da fraction increased slightly (8.51% at 4 h to 10.30% at 10 h), reflecting minor continued proteolysis but without meaningful redistribution of the overall peptide size profile. This stabilization in MW distribution from 4 h onward mirrors the plateau observed in DH and bioactive activities. As previously reported [37], balanced MW distributions enriched in low- to medium-molecular-weight peptide fractions are associated with optimal bioactive peptide profiles.

The plateau in hydrolysis efficiency beyond 4 h can be attributed to progressive substrate depletion, product inhibition by accumulated peptides, and potential reduction in enzyme activity at extended exposure times, as documented by Wu et al. [38]. These observations are consistent with findings by Nongonierma and FitzGerald [37], who noted that prolonged hydrolysis rarely improves the functional properties of protein hydrolysates, and by Arrutia et al. [36], who demonstrated that protein unfolding and enzyme accessibility reach optimal levels within comparable timeframes. Taken together, the convergence of kinetic, structural, and bioactivity data firmly establishes 4 h as the optimal hydrolysis time for HBN-8, and this condition was carried forward for subsequent fractionation, peptide identification, and gastrointestinal stability studies. It should be noted that the hydrolysis conditions in this study were optimized using a one-factor-at-a-time (OFAT) sequential approach. This strategy was deliberately adopted as a screening-stage optimization to identify the most influential substrate–enzyme combination and operating window for goat blood, a substrate not previously characterized for dual-function peptide production. While OFAT is widely applied in food protein hydrolysis research and yielded a robust optimum (HBN-8, 4 h) with strong dual ACE/DPP-IV inhibitory activity, it does not capture potential interactions among enzyme type, substrate concentration, and hydrolysis time. Future studies will therefore employ statistically based experimental designs—such as response surface methodology (RSM) or full-factorial design—to model these interactions, refine the optimum, and support pilot- and industrial-scale process translation.

### 3.3. Fractionation and Purification of Bioactive Peptides

#### 3.3.1. Ultrafiltration Fraction and Bioactivity Profiling

Ultrafiltration (UF) is a widely adopted membrane separation technique for concentrating and purifying bioactive peptides, offering practical advantages at both laboratory and industrial scales [35]. Building on the optimized HBN-8 condition (4 h hydrolysis) identified in Section 3.2, the hydrolysate was subjected to sequential UF fractionation using membranes with molecular weight cut-offs (MWCO) of 10 and 3 kDa, yielding three distinct fractions: UF1 (>10 kDa), UF2 (10–3 kDa), and UF3 (≤3 kDa).

**Figure 2:**
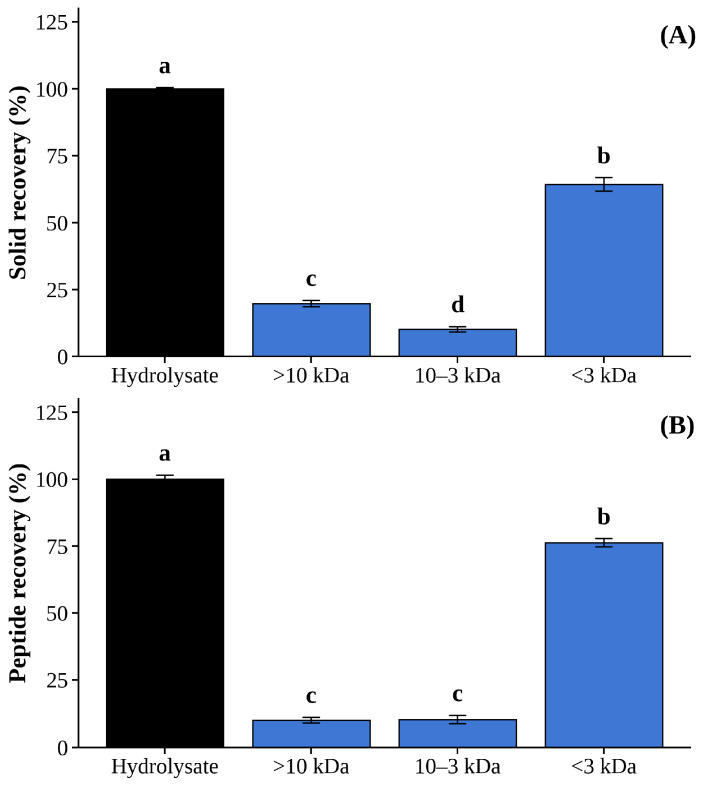
Presented in percentage of solid recovery (**A**) and peptide recovery (**B**) of HBN-8 hydrolysate following ultrafiltration separation into three size-based fractions: greater than 10 kDa (UF1), between 10 and 3 kDa (UF2), and ≤3 kDa (UF3). Bars sharing different letters indicate statistically significant differences within each parameter (*p* < 0.05).

The UF3 fraction (≤3 kDa) accounted for the majority of both solid recovery (~65%) and peptide recovery (~75%), while UF1 (>10 kDa) and UF2 (10–3 kDa) each contributed approximately 10% of total peptide recovery (Figure 2A,B). This distribution is consistent with the MW profile established in Section 3.2.3 (Figure 1), where peptides in the 204–400 Da range predominated following 4 h of Neutrase hydrolysis, confirming that the majority of generated peptides fall within the ≤3 kDa range. The predominance of low-MW peptides in the UF3 fraction aligns with findings from Wu et al. [38], who reported optimal bioactivity in low-molecular-weight peptide fractions from enzymatic hydrolysates.

Enzyme inhibition assays confirmed that UF3 exhibited the highest dual bioactivities among all fractions, with DPP-IV and ACE inhibition of 87.8 ± 1.3% and 65.5 ± 2.1%, respectively (Table 6). Notably, even the intact hydrolysate and UF2 showed substantial DPP-IV inhibition (79.4% and 81.2%), while UF1 (>10 kDa) showed the lowest activities for both DPP-IV (74.9%) and ACE (52.3%), indicating that larger peptides contribute less to the observed bioactivities. This inverse relationship between peptide size and bioactivity corroborates findings by Nongonierma and FitzGerald [37], who demonstrated that peptides below 3 kDa typically exhibit enhanced inhibitory properties due to better steric accessibility to enzyme active sites. Similar size-dependent bioactivity patterns have been reported in hydrolysates from Antarctic krill [39], yellowfin tuna viscera [40], and silver catfish protein [41].

The superior performance of UF3 in both peptide yield and dual bioactive properties collectively establishes it as the most promising fraction for further characterization. As emphasized by Li-Chan et al. [42] and Udenigwe and Aluko [35], short peptides in this size range frequently harbor specific sequence motifs responsible for target biological activities. Therefore, UF3 was selected for subsequent size exclusion chromatography, peptide sequencing, and structural identification to elucidate the sequence–function relationships underlying its dual ACE and DPP-IV inhibitory activities.

#### 3.3.2. Peptide Sequence Identification and in Silico Gastrointestinal Digestion

**Figure 3:**
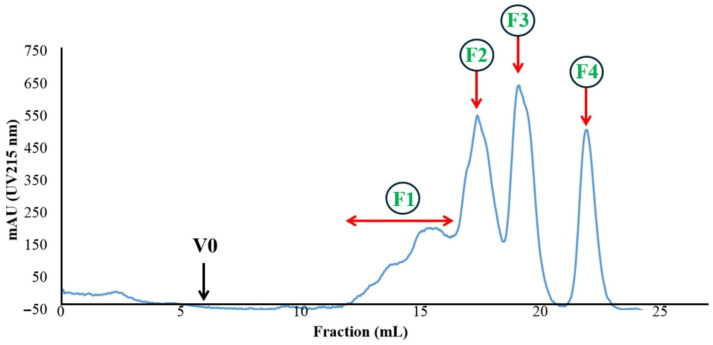
Size exclusion chromatography (SEC) profile of the UF3 fraction (≤3 kDa) from HBN-8, monitored at UV 215 nm. F1–F4 indicate collected sub-fractions; V0 denotes the void volume.

Building on the superior bioactivity of the UF3 fraction (≤3 kDa) established in Section 3.3.1, further purification by size exclusion chromatography (SEC) resolved UF3 into four distinct sub-fractions (F1–F4), as shown in the chromatogram (Figure 3).

F3 accounted for the highest peptide yield (36.19 ± 0.69%) among all SEC fractions, followed by F4 (27.67 ± 0.91%), F2 (25.43 ± 0.24%), and F1 (11.02 ± 0.25%) (Table 7). Enzyme inhibition assays revealed that F3 also exhibited the most potent dual bioactivities, with IC_50_ values of 0.89 ± 0.09 and 0.45 ± 0.03 mg Leu eq./mL for DPP-IV and ACE inhibition, respectively—significantly lower than F1 (3.02 and 5.02), F2 (2.43 and 0.93), and F4 (9.67 and 0.67). Notably, while F4 showed relatively good ACE inhibition (IC_50_ = 0.67 mg Leu eq./mL), its DPP-IV inhibitory potency was markedly poor (IC_50_ = 9.67 mg Leu eq./mL), indicating a lack of dual functionality. F3 was therefore selected for LC-MS/MS peptide sequencing.

LC-MS/MS analysis using de novo sequencing (PEAKS Studio, score >90%) identified 14 novel peptide sequences from the F3 fraction, ranging from four to nine amino acids with molecular weights of 547.61–1175.32 Da (Table 8). This size range falls within the optimal window described by Li-Chan et al. [42] for peptides with enhanced bioavailability and enzyme-inhibitory activity. All identified peptides were confirmed as non-toxic by in silico toxicity prediction, supporting their safety for potential food application. Cell-penetrating peptide prediction (CPPpred scores: 0.15–0.60) and bioactivity ranking (Peptide ranker scores: 0.07–0.79) provided additional in silico evidence of their functional potential, with RMQFR (Peptide ranker: 0.78; CPPpred: 0.60) and WFTQR (0.79; 0.39) scoring highest, suggesting favorable bioavailability characteristics [42].

In silico gastrointestinal digestion using pepsin, trypsin, and chymotrypsin (DFBP database) predicted that 12 of the 14 sequences would generate bioactive fragments upon GI processing (Table 8). Several sequences yielded fragments with dual ACE and DPP-IV inhibitory activities of particular significance. HPLPQTK generated the fragment PL (BIOPEP IDs: 7513, 8638), a proline-containing dipeptide with both ACE- and DPP-IV-inhibitory properties. Proline-containing sequences are well-established for their resistance to digestive proteases and enhanced stability during gastrointestinal transit [43]. REYATAVNK yielded EY (BIOPEP IDs: 7752, 8777), another dual ACE/DPP-IV inhibitor, alongside the novel fragment ATAVN, which has not been previously reported in the BIOPEP database. Similarly, TTKVMMDAK generated VM (BIOPEP IDs: 9882, 8923) with dual inhibitory activities, and NPWETLEMR produced PW (BIOPEP IDs: 8865, 8190) with DPP-IV inhibition and antioxidant properties.

Of notable interest is LHKNK, which was predicted to be resistant to all three simulated digestive enzymes, suggesting this intact sequence may survive gastrointestinal transit and potentially exert its bioactivity directly. Additionally, MLER, LFMER, and EAQLFER all generated the fragment ER (BIOPEP ID: 9944) upon GI digestion, confirming ACE-inhibitory potential across multiple parent sequences. Beyond direct BIOPEP matches, these fragments also align with established structural motif rules for ACE and DPP-IV inhibitors: C-terminal proline or hydrophobic/aromatic residues (Pro, Phe, Tyr, Trp) characteristic of potent ACE inhibitors are present in PL, PW, EY, and VM [44], while N-terminal hydrophobic residues (Leu, Met, Trp) and proline-containing motifs characteristic of DPP-IV inhibitors are evident in LHKNK, LFMER, MLER, and PW [15]. Several fragments, including PQTK, ATAVN, SPQTK, TQR, DL, QSK, TH, QSQL, ETL, EM, TTK, DAK, and EAQL, have no existing BIOPEP records and represent potentially novel bioactive sequences warranting experimental validation.

The size of predicted fragments (2–5 amino acids) is particularly significant for intestinal absorption. Di- and tripeptides can be efficiently transported via the PepT1 transporter system, while longer fragments (4–6 amino acids) may utilize paracellular or transcytosis pathways [45,46]. This suggests that the bioactive fragments identified here could reach systemic circulation via multiple mechanisms of absorption. The stability of key sequences during simulated digestion, combined with their established or predicted transport capabilities, is consistent with previous reports on short-circulating peptides from wheat germ hydrolysate [47] and the antihypertensive β-casein peptide HLPLP [47], and with the comprehensive analysis by Jahandideh et al. [48] on glucoregulatory food-derived peptides.

It should be acknowledged that chemical synthesis and direct enzyme inhibition assays of the individual peptides were not performed in the present study—an explicit limitation. Nevertheless, the structural concordance of the identified sequences with both BIOPEP entries and established ACE/DPP-IV motifs strengthens the predicted dual-function bioactivity, with synthesis-based validation of LHKNK and other top candidates identified as priority future work. Collectively, the identification of multiple non-toxic, dual-function peptide sequences with favorable in silico physicochemical profiles, predicted GI stability, and known intestinal transport mechanisms provides strong foundational evidence for the potential in vivo efficacy of HBN-8 hydrolysate as a functional food ingredient targeting both hypertension and type 2 diabetes.

### 3.4. Gastrointestinal Stability and Bioactivity of HBN-8 Hydrolysate

The gastrointestinal stability of bioactive peptides is a critical determinant of their therapeutic efficacy after oral administration, as peptides must survive sequential proteolytic environments before reaching their target sites [16]. In this study, the stability and bioactive modifications of HBN-8 hydrolysate (4 h) were evaluated during simulated gastrointestinal digestion following the standardized INFOGEST static digestion protocol [26], with monitoring through sequential gastric (pepsin, pH 3.0, 0–2 h) and intestinal (pancreatin, pH 7.0, 2–4 h) phases (Table 9).

Total α-amino content increased progressively throughout digestion in the HBN-8 hydrolysate, from 6.07 ± 0.50 mg Leu eq./mL (undigested) to 12.24 ± 0.67 mg Leu eq./mL (gastric phase) and 16.18 ± 0.57 mg Leu eq./mL (intestinal phase), indicating continued but controlled proteolytic activity generating additional free amino groups. The GI enzyme control showed minimal α-amino release (0.10 and 6.73 mg Leu eq./mL at gastric and intestinal phases, respectively), confirming that the increases observed in the hydrolysate reflect genuine peptide bond cleavage rather than enzyme autolysis. This progressive increase in α-amino content, rather than a plateau, suggests that the hydrolysate still contains susceptible peptide bonds that are gradually cleaved during digestion, consistent with our MW distribution data showing a residual proportion of medium-sized peptides (1000–3000 Da; ~41%) in HBN-8 at 4 h.

Remarkably, both DPP-IV and ACE inhibitory activities were substantially enhanced following GI digestion compared to the undigested sample. DPP-IV inhibition increased significantly from 34.08 ± 1.97% (undigested) to 58.75 ± 6.00% after the gastric phase, though it slightly declined to 53.87 ± 6.88% following the intestinal phase—a difference that was not statistically significant (*p* > 0.05), suggesting that DPP-IV inhibitory activity was largely established during gastric proteolysis and maintained through intestinal digestion. In contrast, ACE inhibitory activity showed a progressive and statistically significant enhancement across all phases, from 35.71 ± 5.77% (undigested) to 56.17 ± 7.78% (gastric phase) and reaching 60.91 ± 2.90% (intestinal phase), indicating that intestinal pancreatin continued to generate ACE-inhibitory fragments. The GI enzyme controls showed substantially lower background inhibition (DPP-IV: 11.16–24.87%; ACE: 18.27–34.48%), confirming that the bioactivity observed in the hydrolysate samples significantly exceeds enzyme-intrinsic effects.

The enhancement in bioactive properties during digestion aligns with our earlier in silico predictions (Section 3.3.2), in which identified parent sequences (4–9 amino acids) were predicted to yield smaller, more potent bioactive fragments (2–5 amino acids) upon GI processing. Fragments such as PL, EY, and VM with dual ACE/DPP-IV activities, and ER with ACE inhibitory properties, are likely released or enriched during this process. The differential response of DPP-IV and ACE inhibition across gastric and intestinal phases further supports the notion that distinct peptide fragments are generated at each digestive stage, with gastric pepsin primarily driving DPP-IV-active fragment release, while pancreatin-mediated intestinal digestion preferentially generates ACE-inhibitory sequences—a pattern consistent with findings reported by Nongonierma and FitzGerald [37].

The stability and enhanced bioactivity can be partly attributed to the hydrolysate’s structural features. The predominance of low-MW peptides (≤3 kDa; ~75% of total peptides) in HBN-8 provides inherent resistance to further extensive proteolytic degradation [35]. Additionally, the presence of proline-containing sequences such as HPLPQTK and FLSPQTK likely confers resistance to digestive proteases, as proline residues are known to impede peptide bond cleavage at adjacent positions [49]. Collectively, these data demonstrate that HBN-8 peptides not only withstand gastrointestinal conditions but are further bioactivated during digestion—a critical characteristic for oral bioactive peptides destined for functional food applications targeting both hypertension and type 2 diabetes management [37,48].

### 3.5. Bioavailability and Retained Bioactivity of Transported Peptides

#### 3.5.1. Cytotoxicity of GI-Digested Peptides Toward Caco-2 Cells

**Figure 4:**
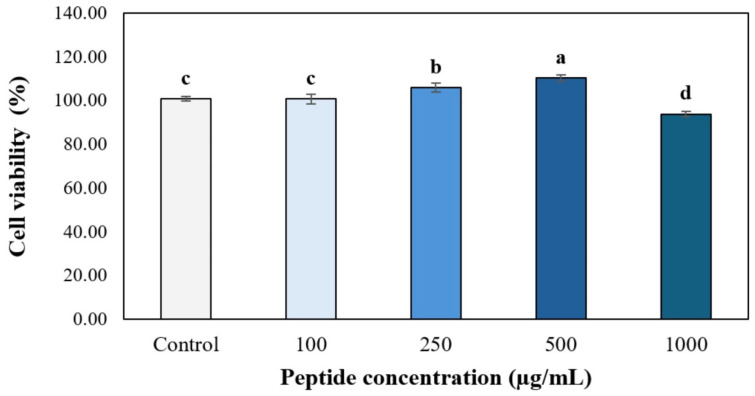
Cell viability of Caco-2 cells following 24 h incubation with gastrointestinal-digested HBN-8 peptides at tested concentrations ranging from 0 to 1000 µg/mL, as evaluated by the MTT assay. Bars sharing different letters ^(a–d)^ differ significantly from one another (*p* < 0.05).

Prior to the transepithelial transport study, the cytotoxicity of GI-digested HBN-8 peptides toward Caco-2 cells was assessed using the MTT assay at concentrations of 100–1000 µg/mL following 24 h incubation (Figure 4). Caco-2 cells are a well-established human intestinal epithelial cell model widely used to evaluate the safety and bioavailability of food-derived bioactive peptides [50]. The results revealed a non-linear, hormetic response across the tested concentration range. At 100 µg/mL, cell viability was not significantly different from the untreated control (*p* > 0.05), indicating no adverse effect at low concentrations. Viability increased significantly above the control at 250 µg/mL (~105%). It reached its highest level at 500 µg/mL (~110%), suggesting that at these concentrations, the peptides may enhance mitochondrial metabolic activity in Caco-2 cells, as reflected by increased formazan production in the MTT assay—a phenomenon previously observed with other food-derived bioactive peptides and potentially attributable to mild stimulation of cellular metabolic pathways [26]. At 1000 µg/mL, cell viability declined to approximately 93–94%, which was statistically significantly lower than the control (*p* < 0.05, letter d). However, it remained well above the 80% cytotoxicity threshold conventionally used to define non-cytotoxic concentrations in food safety assessments.

All tested concentrations, therefore, met the non-cytotoxicity criterion (>80% viability), confirming the safety of GI-digested HBN-8 peptides toward intestinal epithelial cells across the entire concentration range evaluated. The 500 µg/mL concentration was selected for the subsequent transepithelial transport experiment, as it yielded the highest cell viability while providing a sufficient peptide load for accurate quantification of transported fractions and their residual bioactivities. These findings are consistent with cytotoxicity profiles reported for other food protein-derived peptides assessed under comparable conditions [26,50], and collectively support the suitability of HBN-8 peptides for functional food applications intended for oral delivery.

#### 3.5.2. Transepithelial Transport and Residual Inhibitory Activities

**Figure 5:**
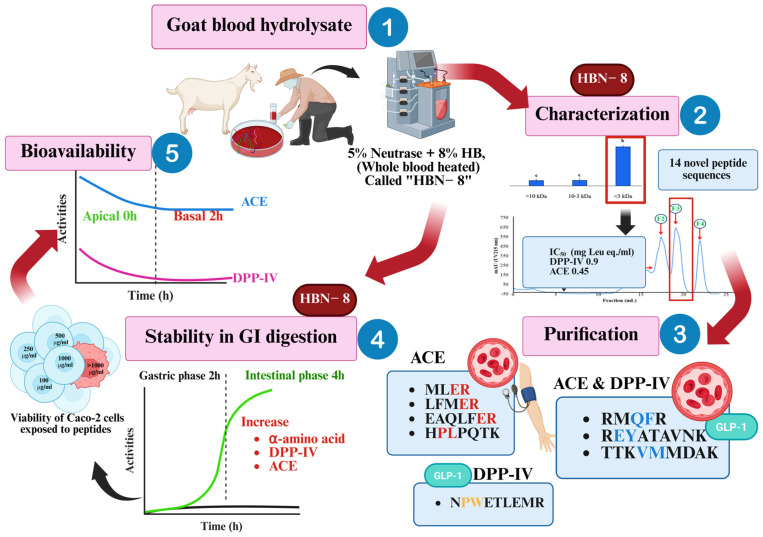
Schematic summary of the valorization of goat blood into dual-function bioactive peptides: from heat pretreatment and Neutrase hydrolysis (HBN-8, 4 h) through ultrafiltration fractionation, LC-MS/MS peptide identification, simulated gastrointestinal digestion, and transepithelial transport assessment using Caco-2 cell monolayers, highlighting key findings for ACE and DPP-IV inhibitory activities.

Following confirmation of GI stability and cellular safety, the transepithelial transport and retained bioactivity of GI-digested HBN-8 peptides were assessed using Caco-2 cell monolayers, with results summarized in Table 10 and the overall study workflow illustrated in Figure 5. Peptide bioavailability across the Caco-2 monolayer was determined to be 10.47 ± 1.09%, representing successful transepithelial transport of a meaningful fraction of GI-digested peptides—a result directly comparable to the 8–12% bioavailability range reported by Vermeirssen et al. [46] for ACE-inhibitory food peptides, and consistent with transport efficiencies reported for goat meat-derived peptides by Luasiri et al. [26] and chicken-derived ACE-inhibitory peptides by Sangsawad et al. [50]. This level of transport is considered functionally relevant for food-derived bioactive peptides, where even modest absorption can produce measurable physiological effects given the relatively high peptide concentrations achievable through dietary intake. Notably, food-derived peptides with comparable transport efficiencies—including the lactotripeptides Val-Pro-Pro and Ile-Pro-Pro—have produced significant antihypertensive effects in both spontaneously hypertensive rat (SHR) models and human clinical trials [10], supporting the functional relevance of this transport range. Apparent permeability coefficients (P_app_) were not calculated because Papp requires the molar concentration of a discrete analyte, whereas UV_215_ nm detection of a multi-peptide hydrolysate quantifies total peptide-bond content rather than individual peptide molarity. Bioavailability was therefore reported as transepithelial peptide recovery (%)—the standard metric for hydrolysate-level Caco-2 assessments [26,50]—with P_app_ determination for purified individual sequences planned as future work.

Critically, the transported peptides (basal fraction, 2 h) retained significant dual inhibitory activities despite the cellular transport process. For DPP-IV inhibition, IC_50_ values increased from 11.29 ± 1.23 mg Leu eq./mL (apical, 0 h) to 15.99 ± 1.70 mg Leu eq./mL (basal, 2 h), representing an approximately 42% increase in IC_50_—or conversely, a retention of ~58% of inhibitory potency. ACE inhibitory activity showed a similar modest reduction, with IC_50_ increasing from 0.72 ± 0.11 to 1.02 ± 0.10 mg Leu eq./mL (~42% IC_50_ increase). Yet, the absolute IC_50_ value of 1.02 mg Leu eq./mL at the basal side remains within a range associated with meaningful biological activity [46,50]. These moderate reductions in potency are attributable to selective transport of peptide subpopulations across the epithelial barrier, as well as possible peptide modifications during transcellular or paracellular transport [48]. Importantly, this degree of activity retention substantially exceeds that reported for many other food-derived bioactive peptides, which commonly lose 50–70% of their inhibitory activity during intestinal transport [48], highlighting the structural robustness of HBN-8-derived peptides.

The maintained bioactivity of transported peptides is mechanistically supported by multiple lines of evidence from earlier sections of this study. The predominance of low-MW peptides (≤3 kDa; ~75%), the proline-rich sequences conferring protease resistance [49], and the in silico-predicted stability of fragments such as PL, EY, ER, and VM during GI digestion (Table 8) collectively explain why a substantial proportion of bioactivity is preserved through both digestion and cellular transport. The convergence between our computational predictions and experimental transport data provides strong cross-validation: fragments predicted to be GI-stable and structurally compact are precisely those most likely to be absorbed via PepT1-mediated or paracellular transport pathways [45,46], contributing to the observed 10.47% bioavailability and retained dual inhibitory activities.

Figure 5 presents a comprehensive schematic overview of the entire study pipeline—from goat blood fractionation and thermal pretreatment, through Neutrase-mediated hydrolysis and MW-based separation, to peptide identification, GI stability assessment, and Caco-2 bioavailability evaluation—visually integrating the key quantitative outcomes at each stage. The workflow highlights how each processing step was systematically optimized to converge on HBN-8 (8% substrate, 4 h, Neutrase) as the lead condition, and how the ≤3 kDa fraction and its constituent peptides demonstrated consistent dual ACE/DPP-IV inhibitory function across in vitro, in silico, and cell-based evaluation platforms. This integrated visual representation underscores the translational trajectory of the research: from slaughterhouse by-product valorization to a characterized, bioavailable, and functionally validated peptide ingredient with potential application in functional foods targeting the co-management of hypertension and type 2 diabetes.

Taken together, the bioavailability of 10.47% and the retained dual inhibitory activity in the transported fraction bridge the critical gap between in vitro bioactivity and potential in vivo therapeutic relevance, positioning HBN-8-derived peptides among the more promising food-derived dual-function bioactive ingredients reported to date.

Among the four blood preparations evaluated, heat-pretreated whole blood hydrolyzed with Neutrase (HBN-8) is recommended for practical use, as it provided the highest peptide recovery and the most potent dual ACE/DPP-IV inhibitory activity while avoiding the additional centrifugation and anticoagulant steps required for HBS, BC, and PM. The HBN-8 hydrolysate could be incorporated as a functional ingredient into protein-fortified beverages, soups, sauces, processed meat products, or encapsulated nutraceutical formulations targeting blood pressure and glycemic control. Future studies should focus on sensory and stability evaluations in real food matrices, pilot-scale production for techno-economic assessment, and animal or human intervention trials to confirm the in vivo efficacy of HBN-8 and its key peptide, LHKNK, on cardiovascular and metabolic health.

## 4. Conclusions

This study valorized goat blood to produce bioactive peptides with dual ACE and DPP-IV inhibitory activities via a systematic four-step optimization. Heat-pretreated whole blood hydrolyzed with Neutrase at 8% protein for 4 h (HBN-8) provided the optimal balance of degree of hydrolysis, protein recovery, and dual bioactivity. Sequential ultrafiltration and size exclusion chromatography enriched the active fraction, from which LC-MS/MS de novo sequencing identified 14 novel non-toxic peptides. Notably, simulated gastrointestinal digestion enhanced—rather than diminished—both ACE and DPP-IV inhibitory activities, while Caco-2 monolayer assays confirmed 10.47% transepithelial transport with retained dual bioactivity and no cytotoxicity up to 1000 µg/mL. These findings establish goat blood as a sustainable and orally bioavailable source of dual-function peptides for the co-management of hypertension and type 2 diabetes. Future work should focus on in vivo validation of antihypertensive and antihyperglycemic effects in spontaneously hypertensive rats and type-2 diabetic models, together with molecular docking of the identified peptides against ACE and DPP-IV. Subsequent translational studies—including pilot- and industrial-scale process engineering, formal sensory evaluation, and regulatory safety-dossier development—will then be required to advance these peptides toward commercial functional food applications.

## Figures and Tables

**Table 1:** Proximate and mineral compositions of goat blood.

Parameters	HB	HBS	BC	PM
**Proximate composition (%)**				
Moisture	78.75 ± 0.14 ^aC^	81.83 ± 0.02 ^aB^	64.21 ± 0.69 ^aD^	93.52 ± 0.02 ^aA^
Protein	20.50 ± 0.41 ^bB^	18.18 ± 0.88 ^bC^	35.7 ± 0.1 ^bA^	6.71 ± 0.47 ^cD^
Ash	1.05 ± 0.23 ^cC^	1.50 ± 0.07 ^cB^	1.61 ± 0.09 ^cB^	1.91 ± 0.09 ^dA^
Fat	0.20 ± 0.02 ^dB^	0.28 ± 0.04 ^dA^	0.17 ± 0.01 ^dB^	0.12 ± 0.01 ^dC^
**Mineral composition (mg/100 mL)**				
Na	71 ± 25 ^eD^	127 ± 44 ^eC^	243 ± 59 ^cB^	655 ± 23 ^aA^
P	263 ± 7 ^cB^	207 ± 5 ^bC^	480 ± 6 ^aA^	75± 2 ^dD^
S	418 ± 10 ^aA^	310 ± 10 ^aB^	400 ± 34 ^bA^	263 ± 9 ^cC^
Cl	280 ± 20 ^bB^	166 ± 5 ^dD^	191 ± 10 ^dC^	400 ± 10 ^bA^
K	261 ± 27 ^cA^	182 ± 6 ^cB^	190 ± 13 ^dB^	76.± 2 ^dC^
Fe	110 ± 4 ^dB^	103 ± 12 ^eB^	148 ± 1 ^eA^	ND
Ca	ND	ND	ND	38 ± 1 ^e^

HB: whole blood; HBS: whole blood with salt; BC: red blood cells; PM: plasma, ND: not detected. Different letters ^a–e,^ and ^A–D^ above the bars indicate statistically significant differences within the column and row, respectively (*p* < 0.05).

**Table 2:** Amino acid composition (g/100 g protein) of goat blood samples. HB: whole blood; HBS: whole blood with sodium citrate; BC: red blood cells; PM: plasma.

Parameters	WHO/FAO/UNU *	HB	HBS	BC	PM
**(** **1) Essential amino acids (EAAs)**					
L-Leucine	5.90	7.89 ^dA^	7.53 ^cB^	6.37 ^eC^	4.45 ^hD^
L-Lysine	4.50	3.75 ^hC^	3.95 ^fB^	2.93 ^kD^	5.38 ^fA^
L-Isoleucine	3.00	7.37 ^dB^	7.13 ^bC^	7.87 ^dA^	7.96 ^cA^
L-Histidine	1.50	8.93 ^cD^	8.84 ^aC^	9.26 ^cB^	12.12 ^aA^
L-Valine	3.90	6.05 ^eA^	5.92 ^dB^	4.39 ^gD^	4.99 ^gC^
L-Phenylalanine	3.80	4.18 ^gD^	4.83 ^dB^	4.71 ^gC^	5.78 ^fA^
L-Threonine	2.30	6.15 ^eA^	5.78 ^eB^	5.84 ^fB^	4.85 ^hC^
L-Tryptophan	0.60	2.83 ^iB^	2.54 ^eC^	1.75 ^lD^	4.52 ^hA^
L-Methionine	1.60	2.54 ^iD^	3.40 ^bC^	4.14 ^iB^	6.26 ^eB^
**Sum EAAs**		**49.71**	**49.92**	**47.27**	**56.31**
**(** **2) Non-essential amino acids (Non-EAAs)**					
L-Alanine	4.15	10.83 ^aA^	5.94 ^cD^	9.63 ^bB^	7.31 ^dC^
L-Serine	3.24	9.59 ^bB^	3.03 ^fD^	10.15 ^aA^	7.17 ^dC^
L-Aspartic acid	4.68	2.98 ^iD^	7.60 ^bA^	4.21 ^iB^	3.70 ^fC^
L-Glutamic acid	9.34	6.44 ^eB^	9.70 ^aA^	6.44 ^eB^	3.08 ^jC^
Glycine	2.49	8.54 ^cC^	4.61 ^dD^	9.03 ^cB^	9.32 ^bA^
L-Arginine	3.87	5.81 ^fB^	4.87 ^dC^	6.50 ^eA^	4.24 ^hD^
L-Proline	3.52	4.10 ^gB^	3.58 ^eC^	4.08 ^jB^	5.01 ^kA^
L-Tyrosine	3.80	1.06 ^jD^	3.24 ^eA^	1.55 ^lC^	2.36 ^lB^
L(-)-Cystine	2.20	0.94 ^jD^	7.10 ^bA^	1.14 ^mC^	1.50 ^mB^
**Sum non-EAAs**		**50.29**	**49.67**	**52.73**	**43.69**
**(** **3) Sum BCAA**		**18.15**	**20.58**	**18.63**	**17.40**

Values sharing different superscript letters within each column differ significantly at *p* < 0.05. Darker shading indicates a higher proportion. * Amino acid requirements of adults (g/100 g protein) estimated by WHO/FAO/UNU (2007).

**Table 3:** Degree of hydrolysis, protein recovery, and bioactive properties of goat blood hydrolysate derived from raw and heat treatments.

Condition	Sample	Degree of Hydrolysis (%)		Protein Recovery (%)		ACE Inhibition (%)		DPP-IV Inhibition (%)	
		Neutrase	Papain	Neutrase	Papain	Neutrase	Papain	Neutrase	Papain
Raw	RHB	15.60 ^aC^	14.92 ^bC^	30.60 ^aD^	30.69 ^aE^	24.9 ^bG^	59.26 ^aE^	27.09 ^bF^	54.79 ^aH^
	RHBS	14.52 ^bD^	15.27 ^aB^	28.03 ^bE^	32.04 ^aC^	18.13 ^bH^	53.10 ^aG^	38.03 ^bE^	64.75 ^aF^
	RBC	10.88 ^bE^	12.12 ^aD^	22.00 ^bE^	26.37 ^aF^	28.46 ^bF^	57.66 ^aF^	56.97 ^aD^	57.50 ^aG^
	RPM	5.29 ^aG^	4.57 ^bF^	11.57 ^aH^	9.21 ^bH^	41.75 ^bE^	70.81 ^aD^	61.79 ^bC^	71.62 ^aD^
Heated	HHB	18.53 ^aA^	16.88 ^bA^	40.28 ^aA^	35.87 ^bA^	88.97 ^aA^	73.07 ^bC^	83.77 ^aB^	66.15 ^bE^
	HHBS	16.14 ^aB^	14.42 ^bC^	35.19 ^aB^	34.25 ^bB^	78.22 ^bD^	86.38 ^aB^	57.04 ^bD^	85.61 ^aB^
	HBC	16.15 ^aB^	15.61 ^bB^	37.11 ^aB^	31.49 ^bD^	82.73 ^bC^	94.30 ^aA^	87.16 ^aA^	89.04 ^aA^
	HPM	7.27 ^aF^	5.39 ^bE^	13.53 ^aG^	13.61 ^aG^	87.59 ^aB^	74.12 ^bC^	58.96 ^bD^	79.39 ^aC^

Enzyme inhibition assays for ACE and DPP-IV were performed with peptides standardized at 1 mg Leu eq./mL in the final reaction volume. HB: Goat blood, HBS: Goat blood precipitate with sodium citrate, BC: Red blood cells; PM: Plasma. Superscript letters ^a–b^ denote statistically significant differences across the row for each parameter, while ^A–H^ indicate significant differences within the same column (*p* < 0.05).

**Table 4:** Degree of hydrolysis, protein recovery, and bioactive properties of goat blood hydrolysate obtained from various substrate concentrations.

Samples	Conditions (Protein Content, g/100 mL)		
	4	8	12
Degree of hydrolysate (%)			
HBN	19.27 ^cA^	26.47 ^aA^	23.60 ^bA^
HBP	17.54 ^bB^	21.08 ^aB^	18.94 ^bB^
BCP	15.17 ^bC^	18.57 ^aC^	17.16 ^aB^
Total protein recovery (%)			
HBN	42.36 ^bA^	45.91 ^aA^	43.56 ^bA^
HBP	34.49 ^cB^	41.67 ^aB^	38.65 ^bB^
BCP	32.43 ^bB^	35.59 ^aC^	33.56 ^bC^
DPP-IV inhibition (%)			
HBN	83.24 ^aB^	83.86 ^aA^	78.33 ^bA^
HBP	74.62 ^aC^	75.15 ^aB^	77.11 ^aA^
BCP	88.84 ^aA^	84.37 ^aA^	78.60 ^bA^
ACE inhibition (%)			
HBN	87.22 ^aA^	86.79 ^aA^	84.62 ^bA^
HBP	65.85 ^bB^	67.23 ^bB^	73.45 ^aC^
BCP	88.17 ^aA^	87.09 ^aA^	81.94 ^bB^

HBN: Heated whole goat blood hydrolyzed with Neutrase; HBP: Heated whole goat blood hydrolyzed with Papain; BCP: Heated red blood cell hydrolyzed with Papain. Enzyme inhibition assays for ACE and DPP-IV were performed with peptides standardized at 1 mg Leu eq./mL in the final reaction volume. Superscript letters ^a–c^ denote statistically significant differences across the row for each parameter, while ^A–C^ indicate significant differences within the same column (*p* < 0.05).

**Table 5:** Total α-amino acid content, degree of hydrolysis, protein recovery, and bioactivities of goat blood hydrolysate peptides derived from the HBN-8 condition.

Hydrolysis Time (h)	Total α-Amino Acid Content (mg Leu eq./mL)	Degree of Hydrolysate (%)	Protein Recovery (%)	DPP-IV Inhibition (%)	ACE Inhibition (%)
0	14.35 ^c^	0.00 ^c^	3.27 ^c^	37.22 ^c^	25.62 ^c^
2	161.50 ^b^	15.50 ^b^	33.36 ^b^	65.63 ^b^	79.93 ^b^
4	298.80 ^a^	28.13 ^a^	44.38 ^a^	81.13 ^a^	88.24 ^a^
6	301.12 ^a^	29.88 ^a^	46.53 ^a^	82.75 ^a^	84.66 ^a^
8	308.15 ^a^	31.14 ^a^	47.51 ^a^	84.43 ^a^	83.46 ^a^
10	314.10 ^a^	32.34 ^a^	48.56 ^a^	83.14 ^a^	85.74 ^a^

Enzyme inhibition assays for ACE and DPP-IV were performed with peptides standardized at 1 mg Leu eq./mL in the final reaction volume. Superscript letters ^a–c^ denote statistically significant differences across the column for each parameter (*p* < 0.05).

**Table 6:** Enzyme inhibitory activities of ultrafiltration fractions obtained from heated goat blood hydrolyzed with Neutrase (HBN-8) at 4 h.

Fraction	Enzyme Inhibition Activities (%)	
	DPP-IV	ACE
Hydrolysate	79.4 ± 2.8 ^b^	59.2 ± 0.1 ^c^
UF1 (>10 kDa)	74.9 ± 0.8 ^c^	52.3 ± 1.1 ^d^
UF2 (10–3 kDa)	81.2 ± 2.2 ^b^	62.1 ± 1.1 ^b^
UF3 (<3 kDa)	87.8 ± 1.3 ^a^	65.5 ± 2.1 ^a^

Enzyme inhibition assays for ACE and DPP-IV were performed with peptides standardized at 1 mg Leu eq./mL in the final reaction volume. Superscript letters ^a–d^ denote statistically significant differences across the column for each parameter (*p* < 0.05).

**Table 7:** DPP-IV and ACE inhibitory and peptide yield (%) of fractions obtained from HBN-8 (UF ≤ 3 kDa) using SEC.

Fractions	Peptide Yield (%)	Enzyme Inhibition Activities (IC_50_ Value, mg Leu eq./mL)	
		DPP-IV	ACE
F1	11.02 ± 0.25 ^d^	3.02 ± 0.25 ^c^	5.02 ± 0.29 ^d^
F2	25.43 ± 0.24 ^c^	2.43 ± 0.24 ^b^	0.93 ± 0.07 ^c^
F3	36.19 ± 0.69 ^a^	0.89 ± 0.09 ^a^	0.45 ± 0.03 ^a^
F4	27.67 ± 0.91 ^b^	9.67 ± 0.91 ^d^	0.67 ± 0.11 ^b^

Values sharing different superscript ^a–d^ letters within each column differ significantly at *p* < 0.05.

**Table 8:** Physicochemical properties, cell-penetrating potential, and toxicity prediction of peptide sequences identified from fraction F3 by de novo sequencing, together with their bioactive fragments and BIOPEP annotations predicted from in silico gastrointestinal digestion.

No.	Identified Peptide	MW	pI	Peptide Ranker	CPPpred	Toxicity	Peptide Fragment After in Silico GI Digestion and Bioactivity *		BIOPEP:ID *
1	MLER	547.67	6.13	0.26	0.57	NT	ER	ACE inhibitor	9944
2	LFMER	694.84	6.13	0.57	0.24	NT	ER	ACE inhibitor	9944
3	RMQFR	736.89	12.48	0.78	0.60	NT	QF	Renin, DPP-IV inhibitor	9431, 8870
4	PFER	547.61	6.13	0.64	0.15	NT	PF	no report	-
5	LHKNK	638.77	10.62	0.10	0.55	NT	Resistant	no report	-
6	HPLPQTK	819.96	10.11	0.35	0.32	NT	PL	ACE, DPP-IV inhibitor	7513, 8638
							PQTK	no report	-
7	REYATAVNK	1051.16	9.75	0.07	0.38	NT	EY	ACE, DPP-IV inhibitor	7752, 8777
							ATAVN	no report	-
8	WFTQR	736.83	11.09	0.79	0.39	NT	TQR	no report	-
9	NPWETLEMR	1175.32	2.54	0.57	0.27	NT	PW	DPP-IV inhibitor, Antioxidant	8865, 8190
							ETL, EM	no report	-
10	TTKVMMDAK	1024.26	9.90	0.07	0.43	NT	VM	ACE, DPP-IV inhibitor	9882, 8923
							TTK, DAK	no report	-
11	NDLLQSK	824.92	6.13	0.11	0.21	NT	DL, QSK	no report	-
12	FLSPQTK	827.97	10.11	0.36	0.18	NT	SPQTK	no report	-
13	EAQLFER	891.97	2.54	0.27	0.20	NT	ER	ACE inhibitor	9944
							EAQL	no report	-
14	THYQSQLK	1012.13	9.75	0.09	0.17	NT	TH, QSQL	no report	-

* Bioactive peptide database: https://biochemia.uwm.edu.pl/biopep/peptide_data.php, accessed on 1 March 2026; Enzymes: pepsin, trypsin, and chymotrypsin were used for in silico GI digestion via http://www.cqudfbp.net, accessed on 1 March 2026. NT means non toxic.

**Table 9:** Total α-amino content, ACE, and DPP-IV inhibition activities of HBN-8 at 4 h before and after passing through GI hydrolysis for 0–4 h.

Conditions	Undigested	Gastric Phase	Intestinal Phase
	0 h	0–2 h	2–4 h
**1.** **Total α-amino content (mg Leu eq./mL)**			
Control (GI enzyme)		0.10 ± 0.07 ^d^	6.73 ± 5.41 ^c^
Goat blood hydrolysate	6.07 ± 0.50 ^c^	12.24 ± 0.67 ^b^	16.18 ± 0.57 ^a^
**2.** **DPP-IV inhibition (%)**			
Control (GI enzyme)		11.16 ± 4.24 ^d^	24.87 ± 2.76 ^c^
Goat blood hydrolysate	34.08 ± 1.97 ^b^	58.75 ± 6.00 ^a^	53.87 ± 6.88 ^a^
**3.** **ACE inhibition (%)**			
Control (GI enzyme)		18.27 ± 4.51 ^d^	34.48 ± 9.71 ^c^
Goat blood hydrolysate	35.71 ± 5.77 ^c^	56.17 ± 7.78 ^b^	60.91 ± 2.90 ^a^

Values sharing different superscript ^a–d^ letters within each activity differ significantly at *p* < 0.05.

**Table 10:** The bioavailability and bioactivity of peptide (HBN-8 at 4 h) obtained from GI digestion were assessed before (referred to as ‘apical at 0 h’) and after (referred to as ‘basal at 2 h’) their transit through Caco-2 cell monolayers.

Parameter	Value
(1) Bioavailability of peptide (%)	10.47 ± 1.09
(2) Bioactivity of peptides (IC_50_ value, mg Leu eq./mL) in the reaction mixture)	
2.1 DPP-IV inhibition *	
- Apical at 0 h	11.29 ± 1.23 ^b^
- Basal at 2 h	15.99 ± 1.70 ^a^
2.2 ACE inhibition *	
- Apical at 0 h	0.72 ± 0.11 ^b^
- Basal at 2 h	1.02 ± 0.10 ^a^

* Values sharing different superscript ^a, b^ letters within each bioactivity assay (DPP-IV or ACE inhibition) differ significantly at *p* < 0.05.

## Data Availability

The original contributions presented in this study are included in the article. Further inquiries can be directed to the corresponding authors.
